# A 3D-printed microhemispherical shell resonator with electrostatic tuning for a Coriolis vibratory gyroscope

**DOI:** 10.1038/s41378-024-00659-8

**Published:** 2024-03-07

**Authors:** Baoyin Hou, Ye Zhu, Chaofan He, Weidong Wang, Zhi Ding, Wen He, Yong He, Lufeng Che

**Affiliations:** 1 College of Information Science and Electronic Engineering, Zhejiang University, Hangzhou, 310027 China; 2 Center for Microelectronics, Shaoxing Institute, Zhejiang University, Shaoxing, 312035 China; 3 State Key Laboratory of Fluid Power and Mechatronic Systems, Zhejiang University, Hangzhou, 310058 China; 4 School of Mechanical Engineering, Zhejiang University, Hangzhou, 310058 China

**Keywords:** Electrical and electronic engineering, Materials science

## Abstract

The emergence of microhemispherical resonant gyroscopes, which integrate the advantages of exceptional stability and long lifetime with miniaturization, has afforded new possibilities for the development of whole-angle gyroscopes. However, existing methods used for manufacturing microhemispherical resonant gyroscopes based on MEMS technology face the primary drawback of intricate and costly processing. Here, we report the design, fabrication, and characterization of the first 3D-printable microhemispherical shell resonator for a Coriolis vibrating gyroscope. We remarkably achieve fabrication in just two steps bypassing the dozen or so steps required in traditional micromachining. By utilizing the intricate shaping capability and ultrahigh precision offered by projection microstereolithography, we fabricate 3D high-aspect-ratio resonant structures and controllable capacitive air gaps, both of which are extremely difficult to obtain via MEMS technology. In addition, the resonance frequency of the fabricated resonators can be tuned by electrostatic forces, and the fabricated resonators exhibit a higher quality factor in air than do typical MEMS microhemispherical resonators. This work demonstrates the feasibility of rapidly batch-manufacturing microhemispherical shell resonators, paving the way for the development of microhemispherical resonator gyroscopes for portable inertial navigation. Moreover, this particular design concept could be further applied to increase uptake of resonator tools in the MEMS community.

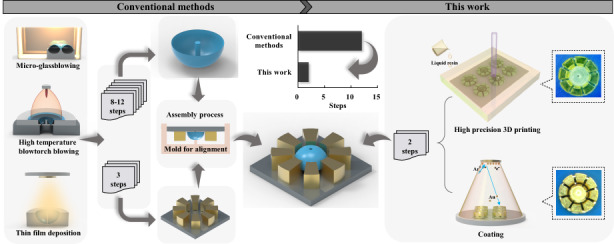

## Introduction

Gyroscopes are essential inertial sensors that are used to measure the rotation angle or angular velocity of an object and are critical components of inertial navigation systems. Traditional gyroscopes have been widely used in fields such as platform stability, aerospace, and inertial navigation^1–4^. Due to the progress in micromachining technology, chip-scale monolithic gyroscopes have been developed to exhibit features such as light weight, small size, and low power consumption, making them suitable for consumer electronics applications such as smartphones^5^.

There are three primary types of gyroscopes: mechanical gyroscopes, optical gyroscopes, and Coriolis vibratory gyroscopes (CVGs). In CVGs, the resonant mass serves as the core sensing element, and its geometry can vary widely; for example, the mass maybe one or more single beam^6,7^, U-shaped tuning forks^8^, butterfly^9–11^, disks^12–19^, rings^20,21^, cylinders^22–24^, or hemispheres. Hemispherical resonator gyroscopes (HRGs) are among the most promising designs for inertial navigation-grade gyroscopes, offering many advantages, such as high sensitivity, low noise and high stability^1,25^. In particular, HRGs can be operated in whole-angle mode due to their ultrahigh quality factor (Q-factor) and complete axial symmetry. Considering the demonstrated advantages and outstanding performance of macroscale HRGs, which are not constrained by any “quantum limitations”^26^, it is inevitable that HRGs will progress toward becoming increasingly smaller sensors to cater to the requirements of upcoming missions. In recent years, much research has focused on miniaturizing HRGs and proposing solutions to achieve wafer-level manufacturability that minimize cost, size, weight, and power consumption^1^. The key problem in miniaturizing HRGs is the fabrication of a microscale hemispherical shell resonator, which is the beating heart of a microhemispherical resonator gyroscope (μHRG).

**Fig. 1:**
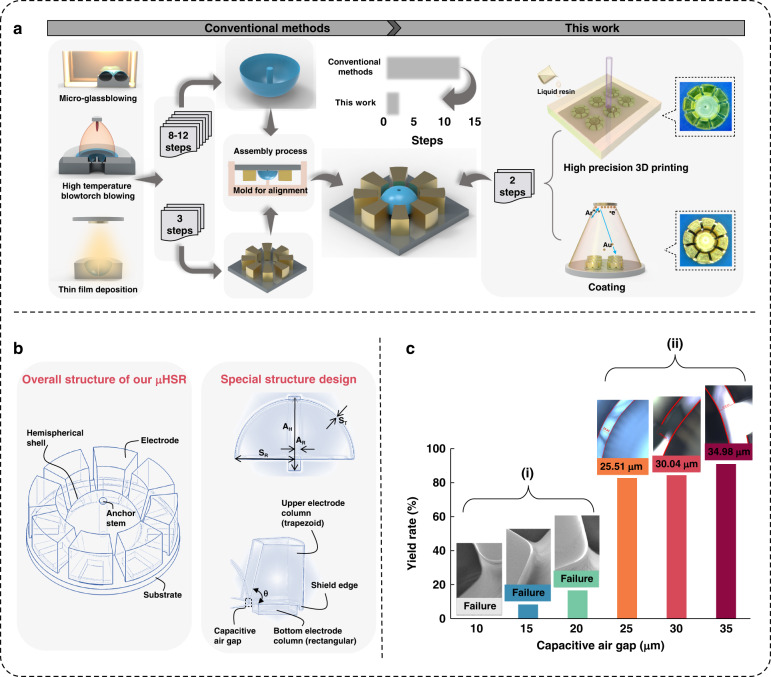
Introduction to the manufacturing methods and mechanical design of microhemispherical shell resonators. **a** An overview of μHSR manufacturing methods. Conventional manufacturing methods typically require 8–12 steps to fabricate the hemispherical shell, approximately 3 steps to fabricate the electrodes, and finally an alignment assembly process to obtain the μHSR. In contrast, our method involves only two steps: finish molding and assembling the hemispherical shell and electrodes. **b** Description of the overall structure of our μHSR. Parameter indications of a hemispherical shell with a central anchor stem and a schematic diagram of a capacitive air gap formed between a single electrode and the hemispherical shell are shown in the partial diagram. **c** Histogram of the yield rate with different capacitive air gaps. The top of each bar chart shows the corresponding capacitive air gap images (the left three insets (i) are taken by SEM showing that the electrodes adhere to the hemispherical shell, and the right three insets (ii) are taken by electron microscopy showing the size of the capacitive air gap)

At present, the conventional manufacturing methods for microhemispherical shell resonators (μHSRs) include microglassblowing^27–31^, high-temperature blowtorch blowing^32,33^ and thin-film deposition^34–39^, as shown in the left half of Fig. 1a. However, these methods have limitations in regard to integrating electrodes for driving and sensing, and the manufacturing costs are high due to the multiple steps and complex fabrication processes involved^40^. To enable the practical use of μHRGs in widespread applications, it is crucial to develop new approaches for simplifying the manufacturing process of μHSR to realize final products that can be practically produced at scale.

The advancement of 3D printing technology enables the fabrication of intricate structures designed with computer-aided design (CAD) software, which is challenging to achieve through traditional micromachining processes^41–43^. Furthermore, 3D printing technology enables the rapid prototyping of products in hours or days, which facilitates batch manufacturing and reduces costs. Recently, various 3D printing techniques have been utilized to fabricate sensors. Liu et al. utilized multimaterial fused deposition modeling (FDM) to create capacitive accelerometers that can detect and monitor human motion^44^. Kamat et al. employed selective laser melting (SLM) to produce flexible piezoresistive microelectromechanical system (MEMS) force sensors that can be integrated into razors^45^.

However, these 3D printing techniques exhibit a minimum resolution ranging from a few tens to hundreds of microns, which limits further miniaturization of the sensors^46^. In contrast, projection microstereolithography (PμSL) has emerged as a preferred option for 3D printing microsensors due to its exceptional printing accuracy, as it offers resolutions down to a few microns or even a few hundred nanometers^43^. These advantages position high-precision 3D printing technology as an increasingly viable alternative to traditional MEMS processes, and some related research has been performed^46–48^.

Here, we present a new approach that enables rapid batch manufacturing of μHSRs, as shown in the right half of Fig. 1a. This approach is based on a precise combination of 3D printing and magnetron sputtering, which involves the integration of molding and surface metallization of the shell and electrodes through special microstructure design. Compared with traditional micromachining methods, this method eliminates need for aligning and assembling hemispherical resonators with electrodes, solves the difficulty in transmitting electrical signals due to material insulation, and, most importantly, reduces more than a dozen fabrication steps down to just two. A μHSR integrated with eight symmetrical capacitive transducers was thus successfully fabricated. Its vibrational and electrical characteristics were measured to validate the approach. The measurement results show that the μHSR features a relatively high Q-factor in air and can be frequency-tuned by manipulating the electrostatic force generated by the voltage across the formed capacitive transducer. In addition, the prototype gyroscope was experimentally analyzed, and its scale factor was obtained. Finally, this approach provides the possibility of manufacturing whole-angle gyroscopes that realize fully matched driving and sensing modes.

## Results

### Mechanical design of the microhemispherical shell resonator

The shape control capability provided by a 3D printing approach allows us to construct a special structural design for μHSR^46,49^. Figure 1b shows the overall structure of our device, which comprises a hemispherical shell with a center anchor stem and eight isolated electrode columns. The anchor stem is exposed 100 μm upwards, forming a cylindrical pad above the hemispherical shell, which is used to guide the intermediate electrode. To obtain a theoretically higher Q-factor, the stem height (*A*_H_), stem radius (*A*_R_), shell thickness (*S*_T_), and shell radius (*S*_R_) were optimized (see Supplementary Note 1 for more details on the optimization); eventually, *A*_H_ = 3.1 mm, *A*_R_ = 0.25 mm, *S*_T_ = 0.05 mm and *S*_R_ = 2.5 mm were selected for appropriate physical parameters of the hemispherical shell resonator.

The eight isolated electrode columns are fabricated radially around the center stem, and each electrode column is divided into upper and lower parts. The upper electrode column is trapezoidal, and the inner side of the column forms a curved slope to realize a smoother, denser metal film during the subsequent sputtering process. The lower electrode column is rectangular, forming an umbrella-like shadow masking the upper part. Through this careful design, the electrode columns can be completely electrically disconnected from each other after metallizing, remarkably forming eight separate electrodes. The height of the lower electrode coincides with the length of the downward exposure of the anchor stem, yielding a precise capacitive air gap between the lip edge of the hemispherical shell and the inner lower edge of the upper electrode.

The size of the capacitive air gap is crucial to the final yield rate of the device. Despite the high optical precision of 2 μm achieved by PμSL, obtaining the appropriate gap size remains a challenging task. We experimented with gap sizes ranging from 10 to 35 µm, with 3 batches of 12 for each gap size. The results are shown in Fig. 1c and a final length of 25 μm was chosen for our device. When the capacitive air gap is less than 25 μm, the yield is less than 20%, and the main reason for this failure is that the electrodes are more likely to stick to the edges of the hemispherical shell, leading to the formation of no capacitive air gap. Although the sample yield rate increases when the capacitive air gap is greater than 25 μm, the initial static capacitance is too small for easy detection. The significance of this experiment lies in the optimization of capacitor gap selection, which leads to improved manufacturing efficiency and device performance. Moreover, these findings underscore the challenges and trade-offs associated with manufacturing at the microscale, providing guidance for the design and production of microscale 3D-printed electronic devices.

### Device fabrication

**Fig. 2:**
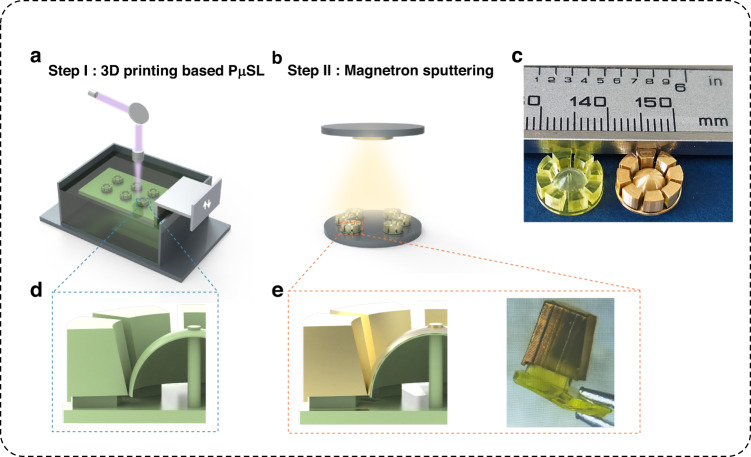
Fabrication and details of μHSRs. **a** Schematic diagram of 3D printing technology. **b** Schematic diagram of the magnetron sputtering process. **c** Comparison of the 3D-printed μHSRs before and after Au sputtering. The scale on the top indicates the size of the μHSRs. **d** Electrode diagram of a 3D-printed μHSR structure. **e** Electrode diagram of μHSRs after magnetron sputtering (3D schematic on the right; physical diagram on the left)

To confirm the feasibility of the abovementioned μHSR device parameters, a two-step fabrication process was employed (Fig. 2a, b). The first step involves using projection microstereolithography (PμSL) equipment to print the μHSR structure, after which a magnetron sputtering system is used to deposit the metal coating and generate the transduction electrodes^46,50^.

In the 3D printing procedure (Fig. 2a), the μHSR structure was established using SolidWorks software (Dassault Systemes, USA) and then sliced into slicing software (BMF Precision Tech, Inc., China)^51^. Subsequently, the digital micromirror device (DMD) on the PμSL equipment is used to control the deflection of each micromirror on the chip according to the two-dimensional data obtained after slicing. The ultraviolet light that is emitted from the LED is reflected by the DMD and then precisely focused onto the surface of the resin material solution (HTL, Young’s modulus 4.2 GPa) through the objective lens. This resin material is selectively exposed on the printing platform to produce a μHSR structure. After the printing of a layer is completed, the platform height is lowered by one layer, and this step is repeated iteratively until all the layers are successfully printed^52,53^.

Reasonably setting the parameters for PμSL can be challenging^54^. Considering the “overcuring” phenomenon in the XY plane^55^, in situations where the capacitive air gap is intentionally made very narrow and exposed for a long period of time, it is possible that the printed air gap may adhere to itself, as shown in Fig. 1b. However, setting a very short exposure time may prevent the formation of microhemispherical shells due to the design parameter of 50 μm thickness. Therefore, it is crucial to strike a balance between the exposure time and the capacitive gap. After optimizing the parameters of the PμSL (details in the “Methods” section), a μHSR with noteworthy features of a 50-μm shell thickness and 25-μm capacitive air gap is achieved and aligns with our target design parameters.

Next, the 3D-printed μHSR structure was treated in a magnetron sputtering system, with a 100-nm Au layer deposited on the various surfaces (Fig. 2c). By employing the design illustrated in Fig. 2d, the deposited Au coating is separated by umbrella-like shadow masking. Figure 2e shows a schematic and physical diagram of the electrodes after sputtering. Notably, the data reveal that the gold coating on the bottom electrode and the substrate is not entirely covered, thereby enabling the eight electrodes to generate distinct potentials.

### Mechanical and vibrational characterization

**Fig. 3:**
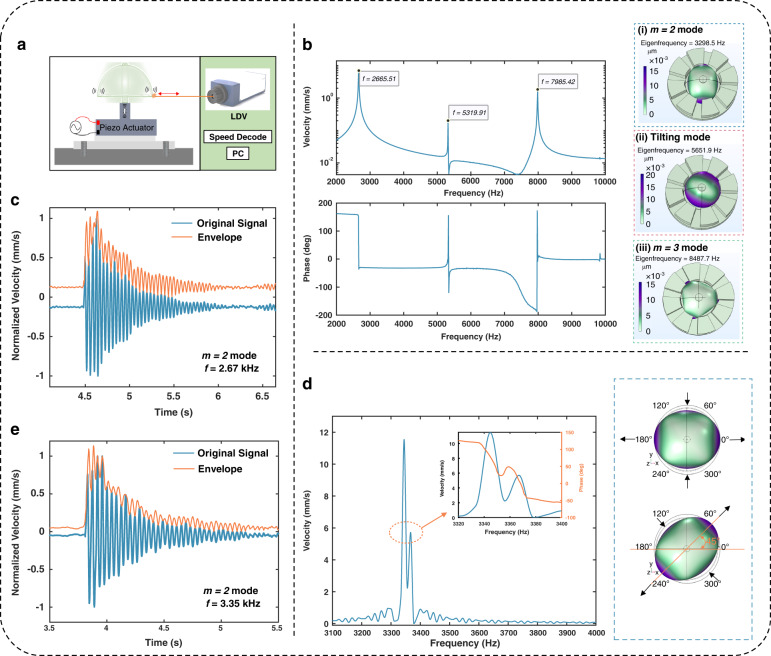
Vibrational characterization of μHSRs. **a** Schematic of the experimental setup for piezoelectric driving and optical sensing of the μHSR. **b** The amplitude and phase spectrum of the μHSR without a metal coating in the range of 2 to 10 kHz, showing different resonance modes and corresponding simulation results of eigenfrequencies. **c** Ring-down plot of the μHSR without a metal coating and its envelope fitting. **d** Frequencies of the $m=2$ degenerate modes and the split ($\Delta f$) between them; the insets on the right show their mode shapes simulated by COMSOL Multiphysics software. To better illustrate the difference between the primary and secondary modes in the direction of vibration, the exact same angle is inserted into the background of the graph. **e** Ring-down plot of the μHSR with a metal coating and its envelope fitting

The mechanical vibration characteristics of the μHSR were measured at room temperature and atmospheric pressure via piezoelectric driving and optical sensing. Figure 3a shows a schematic of the experimental setup (see Supplementary Fig. S2 for a physical diagram of the optical testing equipment). The sinusoidal driving signal from the signal generator is amplified by the power amplifier to stimulate the lead-zirconate-titanate (PZT) piezoelectric actuator. The μHSR is positioned above the piezoelectric shaker, and the shell is driven into resonance by mechanical coupling to the shaker. Then, a high-performance single-point laser vibrometer comprising an optical sensor head and a velocity decoder is used to detect the vibration signal of the device, and the resulting signal is uploaded to a personal computer (PC) for processing.

The laser is pointed at the lip of the hemispherical shell while detecting the deformation, which requires favorable optical reflection of the lip. To improve the light reflectoin, a reflective film with an area slightly larger than the laser spot was stuck to the lip of the shell while measuring the μHSR prior to sputtering the metal coating.

The vibrational spectra are recorded by actuating the piezoelectric shaker with a sinusoidal chirp signal in the specific frequency range of interest and evaluating the device response with fast Fourier transform (FFT) techniques^48^. Figure 3b shows the frequency spectrum of the μHSR after the FFT and vibration modes are simulated by the finite element method (FEM). Three resonance peaks are determined: the $m=2$ mode at 2665.51 Hz (3298.5 Hz simulated), the tilting mode at 5319.91 Hz (5651.9 Hz simulated), and the $m=3$ mode at 7985.42 Hz (8487.7 Hz simulated). The simulation results of the corresponding modes are always larger than the measurements due to the effect of air damping (additional details on the calculations and simulations of resonant frequencies are provided in Supplementary Note 2 and Supplementary Fig. S3). In addition, ring-down plots of the μHSR without and with metal coating were acquired after linear normalization processing with MATLAB (Fig. 3c, e). The Q-factor can be readily determined from^56,57^

$$Q=\frac{\pi \tau }{T}1$$

where ${\rm{\tau }}$ is extracted by fitting the vibration envelope and ${\rm{T}}$ is the resonant period.

In the current μHRG design, the $m=2$ mode, which is also known as the wineglass mode, is selected as the working resonance mode^35,58,59^. To further analyze the frequency behavior of the μHSR with the sputtered metal coating near its $m=2$ mode, a detailed frequency scan is conducted, and the results are illustrated in Fig. 3d. The insets depict the simulation results, which show that the two degenerate modes differ by 45° in the vibration direction. This characteristic is consistent with the expected response of the $m=2$ wineglass mode^32^.

**Fig. 4:**
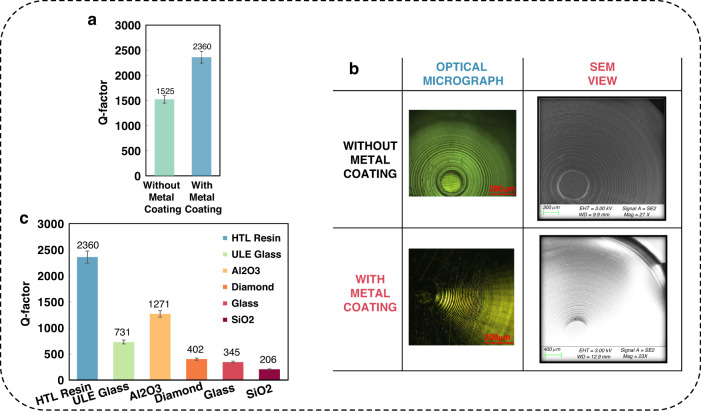
Q-factor analysis of μHSRs. **a** Comparison of the Q-factors between the metal-coated and uncoated μHSRs. **b** Step pattern images of the shell surface before and after sputtering. **c** Histogram of Q-factors extracted from the literature for different μHSRs at room temperature and atmospheric pressure (details and references are reported in Supplementary Table S1 and Supplementary Note 3). The Q-factors of μHSRs are divided according to the material of the shell

A higher Q factor is observed in the μHSR with a metal coating than in the presputtered μHSR (Fig. 4a). The metal coating of the μHSR provides two advantages that may account for the higher Q-factor. One is its favorable optical reflection effect, which means that it is not necessary to adhere the reflective film to the shell during laser testing of the μHSR with a metal coating. The coupling between the reflective film and the shell increases the energy dissipation, resulting in a low Q factor. The other advantage pertains to surface roughness. It is well demonstrated that surface roughness can scatter acoustic waves when the roughness dimensions are on the order of the acoustic wavelength, which can limit the Q-factor of surface acoustic wave devices and other high-frequency resonators^38^. Thermoelastic dissipation in µHSRs originating in the bulk of the shell and near its surfaces due to roughness was investigated in ^60^. The 3D-printed hemispherical shell surface will exhibit a step pattern, which will improve after sputtering (Fig. 4b).

Nonetheless, both the metal-coated and uncoated μHSRs achieved relatively high Q-factors and ${\rm{\tau }}$ values in air without any balancing or tuning after manufacturing. Figure 4c compares the Q-factors of our fabricated μHSR with those reported in the literature that were also obtained experimentally in air. The device with the best performance in terms of a high Q-factor is reported as the first blue bar. While this effect might seem surprising, the strength of the approach is derived from employing the more flexible 3D printing method. The structural design parameters could quickly be adjusted, including the geometry and dimensions of the resonator, to compensate for the shortcomings of the material; such adjustments are difficult to achieve using traditional fabrication methods.

### Electrical properties

**Fig. 5:**
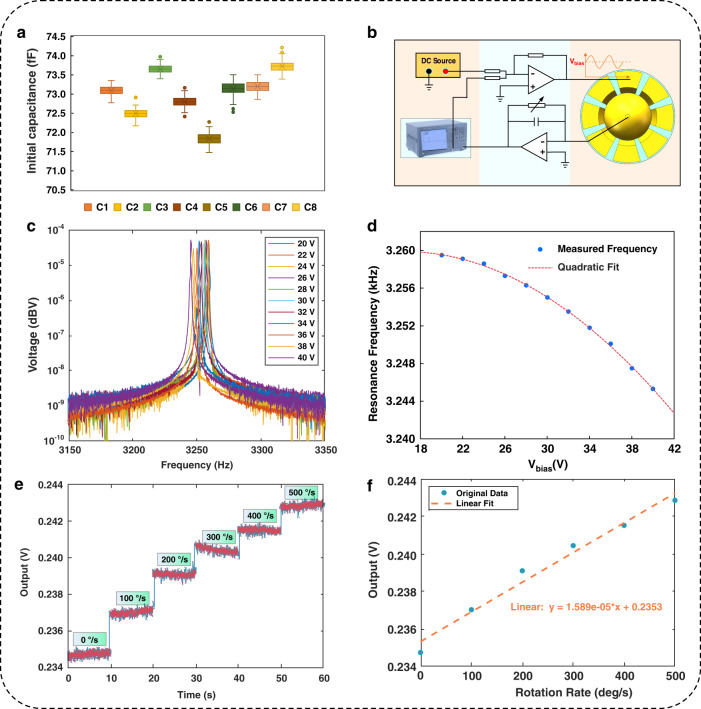
Electrical properties of μHSRs. **a** Box plot of the surrounding eight capacitors (carrier frequency: 10 kHz, sampling rate: 10 Hz, sampling time: 10 s). **b** Illustration of the electronic setup for resonance frequency measurements. **c** The $m=2$ resonance peaks of the μHSR, measured at V_bias_ from 20 V to 40 V. **d** The frequency tuning curve of the $m=2$ mode resonance of the μHSR by changing the electrostatic force, fitted with a quadratic equation. **e** Variation in the output voltage at different rotation rates with a sampling time of 10 s. **f** Open-loop scale factor measurement and fitting results of the μHRG

A substrate with electrodes, including both central anchor and frame bonding areas, can be integrated with a hemispherical shell structure to enable capacitive gyroscope operation^61^. Therefore, relevant electrical performance measurements were performed to validate the usability of the fabricated resonator for μHRGs. The μHSRs with a metal coating were placed into a wafer prober (as shown in Supplementary Fig. S4), and the functional and electrical parameters were directly and conveniently tested. Little difference in the initial capacitance was observed between the eight capacitors formed between the shell and surrounding electrode columns (Fig. 5a), further demonstrating the high structural symmetry and small manufacturing error of the 3D-printed μHSR. The symmetry of the resonance frequencies of the different electrodes is even more critical because the μHSR operates in vibrational modes when used as a gyroscope. Therefore, we measured the resonance frequency of different electrodes using the setup shown in Fig. 5b. The center pad was used as the readout pin, and the driving voltage was subsequently applied to the surrounding electrodes. The results are shown in Supplementary Fig. S5, and the resonance frequencies of the different electrodes exhibit only 0.18% error.

To complete the electrostatic excitation and detection experiments shown in Fig. 5b, the electrodes of the μHSR need to be led out. Although wire bonding is an efficient method for establishing this type of electrical connection in microelectronics, it could lead to the peeling off of the metal coating in the final device (as depicted in Supplementary Fig. S6). Therefore, conductive silver paint was chosen to achieve a wire bond between the electrodes of the μHSR and the contact pad of the prefabricated PCB. During this procedure, the elevated section of the central anchor functions as a pad for the connection of the intermediate electrode, featuring a small surface area. Consequently, an exceedingly thin gold wire must be used to lead out the intermediate electrode and ensure mass symmetry in the hemispherical shell (despite uneven application of conductive silver paint), as depicted in the upper right inset of Supplementary Fig. S7. Fine wires are better suited for the detection of minute signals, and their application at the detection end allows us to achieve the requisite sensitivity. In contrast, thicker wires can be employed to connect the surrounding electrodes, which is more suitable for applying the driving voltage.

Finally, the frequency tuning of our device is achieved by controlling the electrostatic coupling force induced by the voltage across the capacitor. The primary frequency of the $m=2$ resonance mode at a bias voltage (V_bias_) ranging from 20 V to 40 V is measured (Fig. 5c). The results are obtained by converting the output from the transimpedance amplifier into voltage signals and subsequently processing them through software written in MATLAB. In this process, to better scrutinize the phenomenon of electrostatic tuning, we deliberately disregard the subfrequency split from the primary frequency of the $m=2$ mode. Additionally, the primary resonance frequency at a V_bias_ of 40 V was automatically acquired by the network analyzer at the same time and is depicted in Supplementary Fig. S8. The figure illustrates a resonance frequency of 3245.3 Hz, accompanied by a Q-factor of 1776.4. Due to the presence of electrostatic forces, the resonance frequency of the $m=2$ mode here is slightly lower than the laser vibration measurement result. In addition, the Q factor was calculated with the network analyzer using the following formula $Q=f/{BW}$: Here, $f$ is the resonance frequency, and ${BW}$ is the 3 dB bandwidth of the resonance peak.

Moreover, the frequency tuning curve fitted with a quadratic equation for the $m=2$ resonance mode is shown in Fig. 5d. A quadratic curve fit was performed to highlight that the mechanical resonance peak originated from the μHSR rather than being an electrical peak resulting from the characterized PCB^62^. To further highlight the potential application of our device as a Coriolis gyroscope, we established a straightforward open-loop gyroscope test system^35^. The experimental site and setup are shown in Supplementary Fig. S9, and a detailed description of the equipment in the system is provided in the “Methods” section. Eventually, the sensitivity of the device to the angular rate, the core functionality of a Coriolis gyroscope, is measured experimentally and presented in Fig. 5e, f. The outcomes revealed a scale factor of 15.89 μV/°/s for this gyroscope.

## Discussion

We have achieved a significant milestone by successfully demonstrating the fabrication of a μHSR with integrated electrodes using 3D printing based on PμSL. Additionally, our innovative microstructure design allows the magnetron sputtering process to metallize the surface of the hemispherical shell and enable electrical disconnection. The proposed integrated capacitive electrodes were fabricated, and its transduction efficacy was verified by electrostatic excitation and detection; this approach facilitates the subsequent integration and packaging of the μHRG. Moreover, two different methods were used to test the resonance frequency and Q-factor of the μHSR, and the results revealed the superior Q-factor (in air) of the μHSRs fabricated by the 3D printing method compared to that of other fabrication methods.

Favorable agreement between the theoretical model and experimental measurements is observed, thus demonstrating the favorable reliability of the proposed design and manufacturing process. The experimental results on the μHSR angular rate response show that our device has the potential to be a functioning gyroscope. Therefore, our μHSRs could inspire unique designs and base technologies for a new generation of small and accurate gyroscopes. Furthermore, by combining 3D printing technology with proven processes based on MEMS through rational structural design, the potential for realizing more advanced 3D micromechanical devices could be unlocked.

## Methods

### Sample fabrication

#### 3D printing process

The optimal printing parameters were established for the PμSL equipment (S130, BMF, China) and included a power density of 10 mW/cm^2^, an LED wavelength of 405 nm, a slice thickness of 50 μm, and an exposure time of 2 s. A rigid resin HTL with a Young’s modulus of 4.2 GPa, purchased from BMF, was used for layer-by-layer printing on the platform. Due to the ultrahigh XY printing accuracy and optimized printing parameters, we were able to obtain a capacitive air gap as small as 25 μm.

#### Post processing

Once the 3D-printed μHSR structure was successfully printed, it underwent a cleaning process in which it was alternately sonicated with 75% alcohol and fluorinated solution. This step effectively removed residual resin solution and impurities from the surface. Afterward, the μHSR was thermally cured and dried in an 80 °C environment for 2 h.

#### Magnetron sputtering process

The 3D-printed μHSR structure was positioned on the bench of the magnetron sputtering system, the sputtering power was 200 W, the sputtering time was 100 s, and the gold deposition rate was 60 nm/min. Finally, a layer of 100 nm thick gold was deposited on different surfaces of the 3D-printed μHSR structure.

### Device characterization

#### Optical measurement technique

The frequency response and decay time of the fabricated μHSR were measured by a laser Doppler vibrometer (LDV OFV-505, Polytec Gmbh) consisting of an optical sensor head and a digital velocity decoder with adaptive digital signal processing (DSP) filtering. The vibrometer operates at DC-350 kHz with an optimal velocity distinguishability of 0.01 μm/s (1 Hz bandwidth), fully meeting the experimental requirements. During the experiment, the presence of electrode columns around the hemispherical shell interfered with the laser hitting the lip edge of the shell; hence, one of the electrode columns was destroyed during the experiment.

#### Electrical

The sputtered μHSRs were first placed on a wafer prober (Summit S12000B - M, Cascade Microtech, Inc.) for preliminary screening, based on the principle of no electrical conduction between adjacent electrodes and the same initial value of the eight capacitors formed. A precision LCR meter (E4980A, Keysight Technologies) was used to measure the conductivity and capacitance. Then, the electrodes of the final device were wired out with conductive silver paint (05001-AB, SPI Supply, USA), which was air dried to a tack-free state within 30 min and achieved adequate conductivity and adhesion after 24 h in air at 25 °C. The device excited electrostatically over a desired frequency range is connected to a network analyzer (E5061B, Keysight Technologies) through a transimpedance amplifier constructing the feedback loop for stable analysis. Since the current induced by the capacitance change is very weak, we used an electrometer (Keithley 6517B, Tektronix, Inc.) to act as the transimpedance amplifier. The test setup for electrostatic excitation and detection can be found in Supplementary Fig. S7. Additionally, Supplementary Fig. S9 shows a physical diagram of our open-loop gyroscope experimental setup, which consists of a turntable, our device integrated with an adapter board, an interface circuit PCB, a data acquisition card and signal sources, including a power supply, a signal generator, and a network analyzer. The interface circuit PCB integrates C/V conversion modules, demodulation modules, and amplification and filtering modules. The signal sources provide the necessary power, high-frequency carriers, and drive signals.

### Supplementary information

Supplementary Information

## Data Availability

All the data are available in the main text or the supplementary materials.
