# Supplementary material for: A 3D-printed microhemispherical shell resonator with electrostatic tuning for a Coriolis vibratory gyroscope
Source: Microsyst Nanoeng. 2024 Mar 7;10:32. doi: 10.1038/s41378-024-00659-8 (PMC10918184; doi:10.1038/s41378-024-00659-8)
Supplement: Supplementary file 1 — Supplementary Information [file 41378_2024_659_MOESM1_ESM.docx]

**SUPPLEMENTARY INFORMATION**

**A 3D-printed micro-hemispherical shell resonator with electrostatic tuning for Coriolis vibratory gyroscope**

**Authors:**

Baoyin Hou^1,2,#^, Ye Zhu^1,#^, Chaofan He^3,4^, Weidong Wang^1^, Zhi Ding^1^, Wen He^3,4^, Yong He^3,4,*^, Lufeng Che^1,2,3,*^

#These authors contributed equally to this work.

*Corresponding author. Email: [*lfche@zju.edu.cn*](mailto:lfche@zju.edu.cn)

*Corresponding author. Email: [*yongqin@zju.edu.cn*](mailto:yongqin@zju.edu.cn)

**This PDF file includes:**

Supplementary Text

Notes (1 to 3)

Figs. S1 to S9

Tables S1

References (1 to 5)

**Supplementary Note 1 – Optimization of structural parameters for higher quality factor**

Higher quality factor (Q) of the micro-hemispherical shell resonator (μHSR) always means higher sensitivity and lower noise for the gyroscope. In resonant devices, the Q is defined as the ratio of the energy stored in the resonator to the energy dissipated during a resonant period, and can be expressed as

$Q=2\pi\frac{E}{\Delta E}=2\pi\frac{E}{\sum_{i=1}^{n} \Delta E_{i}}=\left( \sum_{i=1}^{n} \frac{1}{Q_{i}} \right)^{-1} \left( 1 \right)$

where $E$ is the total energy stored and $\Delta E$ is the energy dissipated (per cycle), $\Delta E_{i}$ is the energy dissipation caused by the ith dissipation mechanisms. $Q_{i}$ is the quality factor corresponding to the ith dissipation mechanism.

The above equation shows that the total Q observes the wooden barrel theory (the minimum quality factor corresponding to various dissipation mechanisms determines the ultimate Q). The dominant energy dissipation mechanisms in our devices are air damping ($Q_{air}$), thermoelastic damping ($Q_{TED}$), support loss ($Q_{support}$) and surface loss ($Q_{surface}$), thus, the total Q can approximately calculate by

$$\frac{1}{Q_{total}}=\frac{1}{Q_{air}}+\frac{1}{Q_{TED}}+\frac{1}{Q_{support}}+\frac{1}{Q_{surface}} \left( 2 \right)$$

- 1. **Air damping (**$\boldsymbol{Q}_{\boldsymbol{air}}$**)**

The air damping ($Q_{air}$) is an irreversible dissipation mechanism caused mainly by collision and friction between air particles and the lip of the μHSR. The contribution of drag force damping due to collisions with air molecules is negligible when considering that the μHSRs will be packaged in a vacuum for subsequent gyroscope fabrication. However, the squeeze film air damping ($Q_{squeeze}$) caused by the squeeze of air film between the lip and the upper electrodes remains an important energy dissipation mechanism. This damping effect persists even in high vacuum conditions due to the narrow capacitive air gap used for driving and sensing the device. Since the μHSR operates at the low frequency and vibrates with a small amplitude, it can be assumed that the air film is incompressible, then the $Q_{squeeze}$ induced by the individual capacitive air gap can be evaluated as

$$Q_{squeeze}=\left( 2\pi\right)^{\frac{5}{2}}\rho ft\left( \frac{h_{0}}{S} \right)\sqrt{\frac{RT}{M}}\frac{1}{P} (3)$$

where $\rho$, $f$, $t$ are the resonator mass density, intrinsic frequency and thickness, respectively, $h_{0}$ and $S$ are the distance and peripheral length of the gap, and $R$, $T$, $M$, $P$ are the molar mass of the gas, temperature, universal molar gas constant and damping pressure of the gas film.

The calculation results have shown that, the squeeze film air damping yields to a negligible Q contribution for our resonators at 100 μTorr vacuum pressure when the gap distance exceeds 20 μm since the value can reach 10^9^.

- 1. **Thermoelastic damping (**$\boldsymbol{Q}_{\mathbf{TED}}$**)**

Thermoelastic damping originates from the interaction between the vibration of the resonator and the thermal transmission. The elastic wave strain field arouses a change in internal energy of the resonator structure, causing an irreversible loss of the thermal energy via heat flow through thermal gradients. To characterize this dissipation, all the thermoelastic control equations distributed over the structure of interest should be solved. Ultimately, the $Q_{TED}$ corresponding to the single relaxation mechanism of the structure can be well approximated as

$$Q_{TED}=\frac{1+\left( \omega\tau_{th} \right)^{2}}{\Delta_{E}\cdot\omega\tau_{\mathrm{th}}} \left（ 4 \right）$$

where $\tau_{\mathrm{th}}$ is the thermal relaxation time constant given by

$$\tau_{\mathrm{th}}=\frac{\rho C_{P}ⅆ^{2}}{\pi^{2}\kappa} \left（ 5 \right）$$

and the thermal relaxation strength $\Delta_{E}$ is defined by

$$\Delta_{E}=\frac{E\alpha^{2}T_{0}}{\rho C_{P}} \left（ 6 \right）$$

Within the above, $\omega$ is angular frequency of structural vibration, $T_{0}$ is the ambient temperature and $ⅆ$ as a function of geometry is the thermal path length. The remaining material properties are the density $\rho$, the Young’s modulus $E$, the linear coefficient of thermal expansion $\alpha$, the specific heat $C_{P}$, and the thermal conductivity $\kappa$.

In order to express more intuitively the influence of the dimensional parameters of the hemispherical shell on the thermoelastic damping, COMSOL Multiphysics is used as the finite element software to analyze the $Q_{TED}$ of the entire structure. Fig. S1a, b show the temperature deviation of the μHSR working at its $m = 2$ resonance mode. They clearly reflect the relationship between the strain field and the temperature field, along with the direction of the flow of thermal energy. Based on the simulation data, it is easy to plot the $Q_{TED}$ versus the radius (r) and thickness (t) of the μHSR at $m = 2$ mode, as shown in Fig. S1c, d.


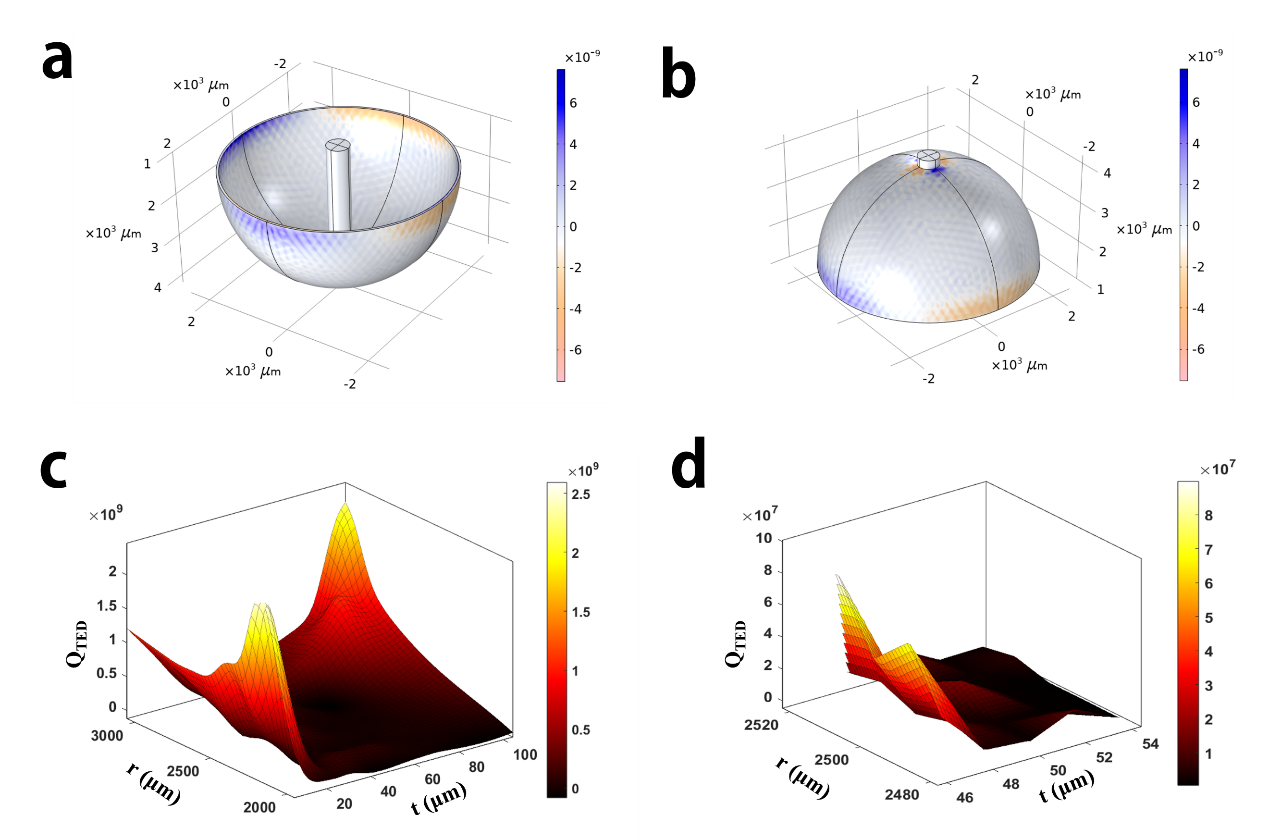


**Supplementary Fig. S1 Simulation results of thermoelastic damping. a** Temperature deviation of the μHSR at the $m = 2$ mode on the front side. **b** Temperature deviation of the μHSR at the $m = 2$ mode on the back side. **c** The plot of $Q_{TED}$ versus radius ($r$ from 2000 to 3000 μm) and thickness ($t$ from 20 to 100 μm) of the μHSR. **d** The plot of $Q_{TED}$ versus radius ($r$ from 2480 to 2520 μm) and thickness ($t$ from 46 to 54 μm) of the μHSR.

- 1. **Support loss (**$\boldsymbol{Q}_{\boldsymbol{support}}$**)**

The $Q_{support}$ is caused by the dissipation of vibration energy in the form of elastic waves, which are partially transferred to the substrate through the support structure. Due to the complexity of the support dissipation mechanism, a perfectly matched layer (PML) method based on finite element analysis has been developed to evaluate the $Q_{support}$ of resonators. The PML absorbs incident waves over a wide range of frequencies for any non-zero incidence angle. In our device, the anchor post fixed to the substrate is subjected to shear forces and moments when it is excited to undergo bending vibrations. Meanwhile, the elastic waves generated by the intrinsic pressure waves entering the shell propagate through the anchor post to the substrate. Therefore, the substrate of μHSR is configured as a PML. Then, all the elastic waves attenuated during the transmission from the support to the PML are analyzed in COMSOL Multiphysics software. The results show that the $Q_{support}$ is mainly influenced by the support radius and length for a certain thickness and radius of the hemispherical shell. By setting the support parameters to those described in the manuscript, the $Q_{support}$ can reach 10^12^, which contributes very little to the total Q.

- 1. **Surface loss (**$\boldsymbol{Q}_{\boldsymbol{surface}}$**)**

The surface loss primarily arises from surface stress, which could be substantially influenced by absorbates on the surface or surface defects. When absorbates are present on the surface, charge transfer occurs between the surface and the absorbates. As a result, the Coulomb repulsion between the dipole moments associated with the absorbate atoms, operating at a close proximity, alters the surface stress and contributes to surface loss. Due to the lack of a complete understanding of this particular energy loss mechanism, there are no reported attempts to model surface loss for estimating the $Q_{surface}$ of the μHSR. However, any portion along the lip of the shell with significant strain can be approximated as a bent beam during the vibration of the μHSR, leading to the expression of its $Q_{surface}$ as follow

$$Q_{surface}=\frac{dt}{2\delta\left( 3d+t) \right.}\frac{E}{E_{ⅆ}} (7)$$

where, $d$, $t$ and $E$ represent the width, thickness and Young's modulus of the beam, respectively. $\delta$ is the thickness of the coating material. $E_{ⅆ}$ related to the surface stress denotes the dissipation value of the Young’s modulus of the surface coating.

Evaluating the surface loss in micro-hemispherical resonator gyroscopes is a challenging endeavor, encompassing various complexities. Achieving precise concurrence between calculated approximations and real-world outcomes remains a persistent challenge, thereby warranting further exploration in future research endeavors.

**
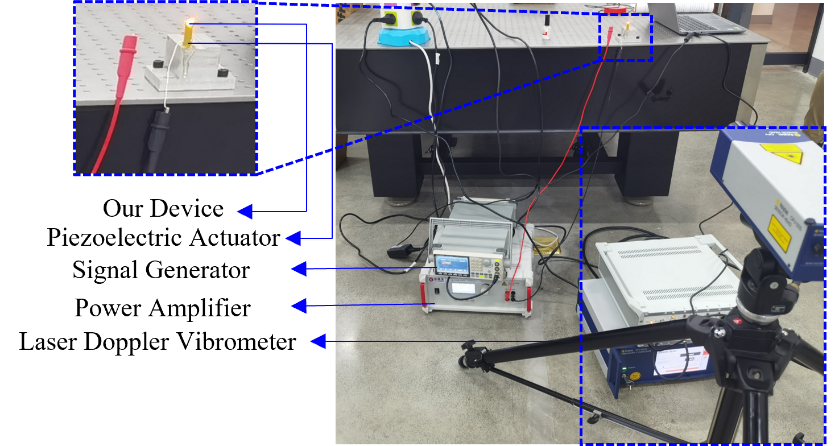
**

**Supplementary Fig. S2** **The physical diagram of optical testing equipment.** During the measurement, the sputtered μHSR looks like a small "light bulb" that is glowing, showing the excellent optical reflection of the laser by the metal coating.

**Supplementary Note 2 –** **Calculation and simulation of resonance frequency**

If the shell thickness is uniform throughout the geometry, natural frequency can be obtained by assuming equal peaks of strain energy and kinetic energy

$$f_{m}=\frac{m\left( m^{2}-1 \right)}{2\pi r^{2}}\sqrt{\frac{E\cdot I\left( m,t \right)}{3\left( 1+\mu\right)\rho\cdot J\left( m,t \right)}} (8)$$

where,

$$I\left( m,t \right)=t^{2}\int_{\varphi_{0}}^{\varphi_{F}} \frac{\tan^{2m} \left( \varphi/2 \right)}{\sin^{3} \varphi}ⅆ\varphi(9)$$

$$J\left( m,t \right)=t\int_{\varphi_{0}}^{\varphi_{F}} \left( m^{2}+1+\sin^{2} \varphi+2m\cos\varphi\right)\tan^{2m} \left( \varphi/2 \right)ⅆ\varphi(10)$$

In the above, $m$ is the order of the natural mode, $r$ is the shell radius, $t$ is the shell thickness, $\varphi_{0}$ and $\varphi_{F}$ are the starting and ending boundary angles, and $E$, $\mu$, $\rho$ are material Young’s modulus, Poisson ratio and density, respectively. The resonant frequencies are simulated in COMSOL Multiphysics software using the FEM, and the results are shown in Fig. S3.


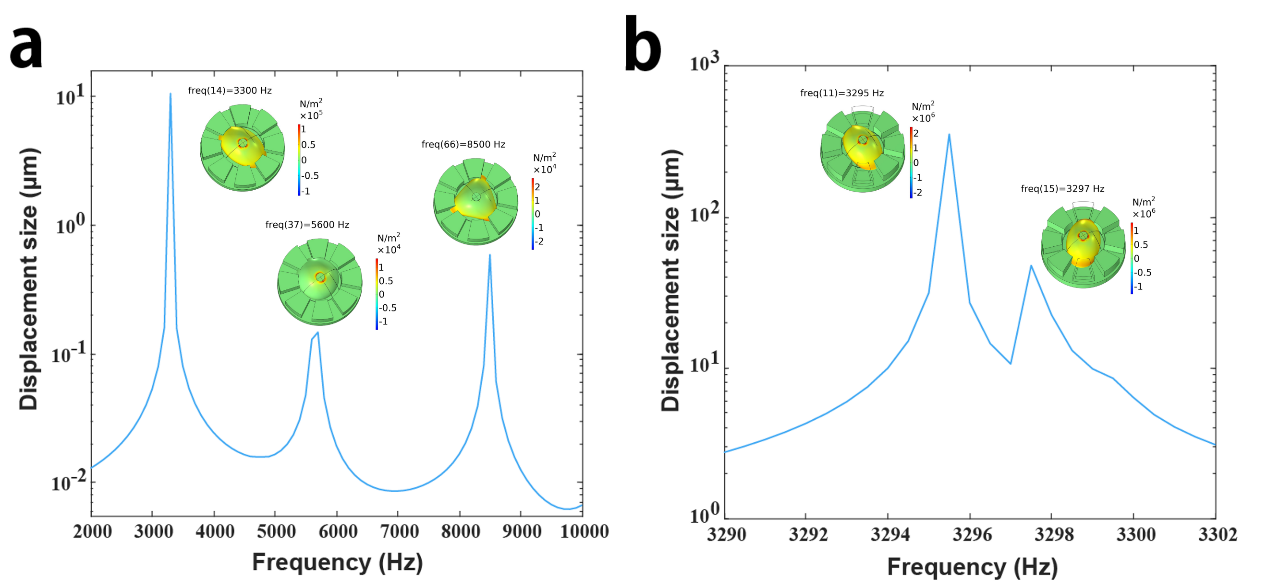


**Supplementary Fig. S3** **The simulated frequency spectrum of the μHSR. a** The simulation result of the frequency response, sweeping from 2 kHz to 10 kHz. The three insets are simulated stress distribution diagrams showing the different resonance modes. **b** Frequency mismatch for the $m = 2$ degenerate modes simulated by FEM. It is observed that although the frequency separation in the degenerate modes is only 2 Hz, the deformation direction of the hemispherical shell differs by an estimated 45°. Two centrally symmetrical electrode columns are hidden in the insets to show the difference in telescoping direction between the degenerate modes.

**Supplementary Note 3 – Q of different μHSRs**

**Supplementary Table S1 Performances for different μHSRs in the air.** The manufacturing methods, materials and response of the μHSRs are also listed in the table. It is observed that the resonance frequency of our device is the lowest, but the Q is the highest. In addition, the relative frequency mismatch of our device of the $m = 2$ degenerate modes is 0.66%, which can be eliminated by electrostatic tuning to achieve mode-matching. Consequently, our device has unique application potential for manufacturing whole-angle gyroscopes.

| **Method** | **Material** | **Resonator Response (m = 2 mode)** | | | **Ref.** | |
| --- | --- | --- | --- | --- | --- | --- |
|  |  | Resonance Frequency  (kHz) | Frequency Mismatch (Percentage of Resonance Frequency) | Q |  |  |
| **3D-SOULE Process** | Glass | 1379 |  | 345 | | Li, Tao. et al. [1] |
| **Micro-mold Machining** | ULE Glass | 5.84 |  | 731 | | Rahman, M. M. et al. [2] |
| **Thin Film Deposition** | Al_2_O_3_ | 60 | 100 Hz (0.17%) | 1271 | | Gray, J. M. et al. [3] |
|  | Diamond | 81.3 | 699.18 Hz (0.86%) | 402 | | Liu, Z Y. et al. [4] |
|  | SiO_2_ | 2200 |  | 206 | | Bhat, A. K. et al. [5] |
| **3D-printing** | HTL resin | 3.35 | 22 Hz (0.66%) | 2360 | | Our work |

**References**

1. Li, T., Visvanathan, K. & Gianchandani, Y. B. A batch-mode micromachining process for spherical structures. *J. Micromech. Microeng.* **24**, 25002 (2013).

2. Rahman, M. M., Xie, Y., Mastrangelo, C. & Kim, H. 3-D hemispherical micro glass-shell resonator with integrated electrostatic excitation and capacitive detection transducers. 2014 IEEE 27th international conference on MEMS. p. 672-675 (2014).

3. Gray, J. M. et al. Hemispherical micro-resonators from atomic layer deposition. *J. Micromech. Microeng.* **24**, (2014).

4. Liu, Z. Y., Zhang, W. P., Cui, F., Tang, J. & Zhang, Y. Y. Fabrication and characterisation of microscale hemispherical shell resonator with diamond electrodes on the Si substrate. *Micro Nano Lett.* **14**, 674-677 (2019).

5. Bhat, A. K., Fegely, L. C. & Bhave, S. A. GOBLIT: a giant opto-mechanical bulk machined light transducer. *Hilton Head 2014*, 247-250 (2014).


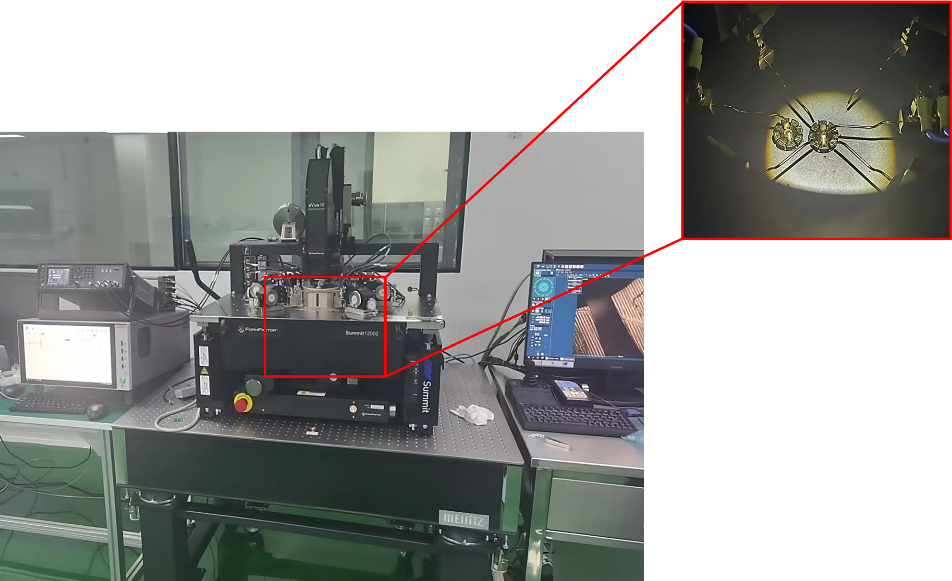


**Supplementary Fig. S4** **Preliminary measurement system diagram of functional and electrical parameters.** The probes are controlled to touch the electrodes of μHSR and then connected to the LCR meter for measurement.


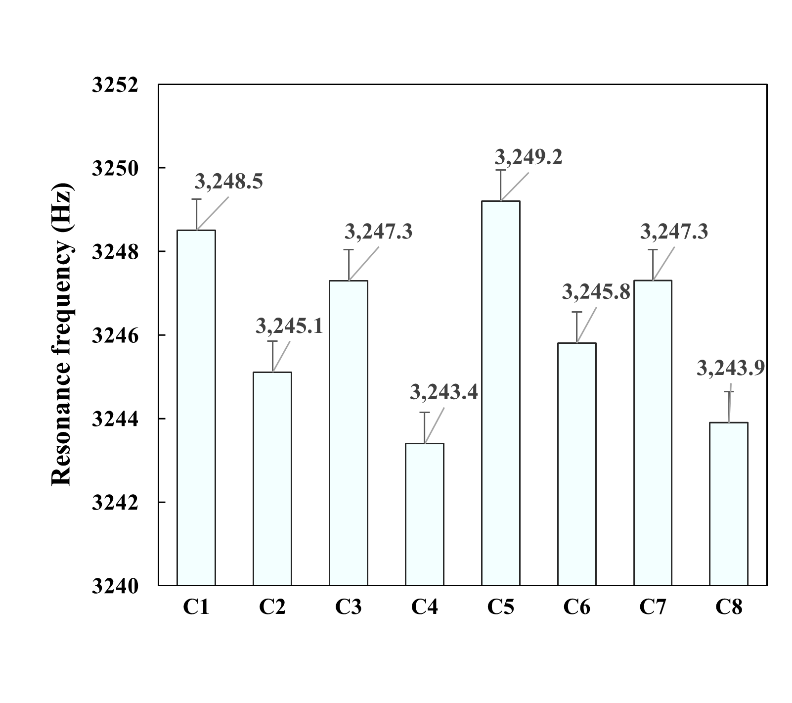


**Supplementary Fig. S5** Resonance frequency measurements of different electrodes (V_bias_ = 40 V).

**
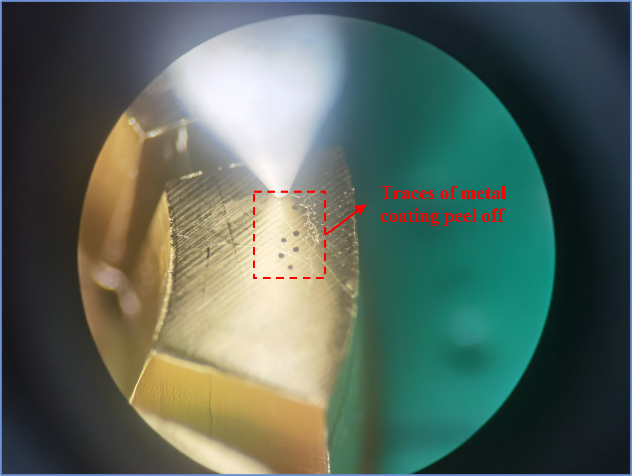
**

**Supplementary Fig.** **S6** **Surface view of a single electrode with detachment traces.** The metal coating peeled off during the use of wire bonding technology based on the traditional micro electromechanical system process.


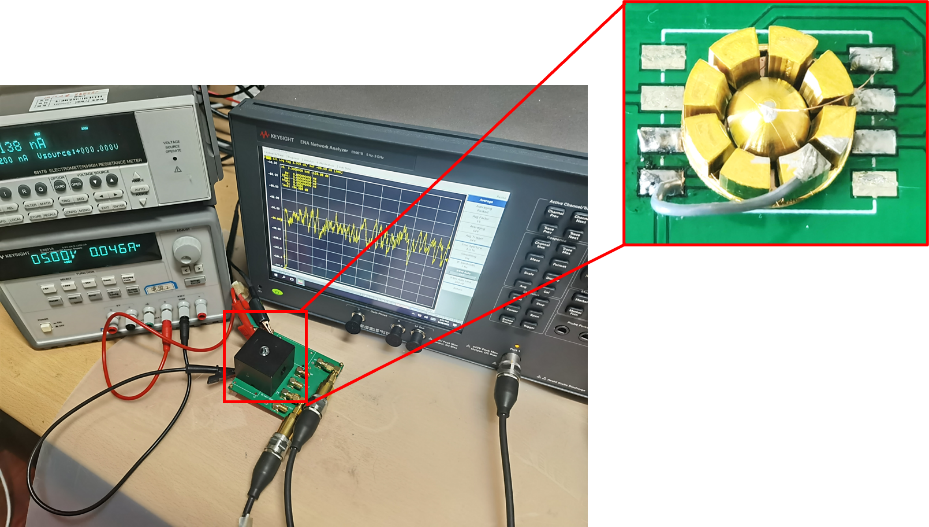


**Supplementary Fig. S7 Testing setup for electrostatic excitation and detection.** Channel 1 of the network analyzer provides the AC-DC superimposed driving signal, and channel 2 serves to detect the sensing signal. The illustration in the upper right corner shows the connection details of the μHSR to pre-fabricated PCB.


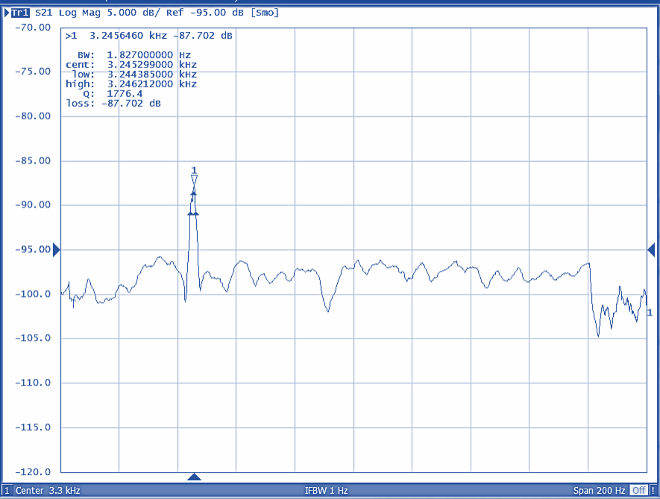


**Supplementary Fig. S8 The frequency response of the μHSR at the V_bias_ of 40 V (center frequency: 3.3 kHz, span: 200 Hz).** The data in the top-left corner of the figure is automatically acquired by the network analyzer.


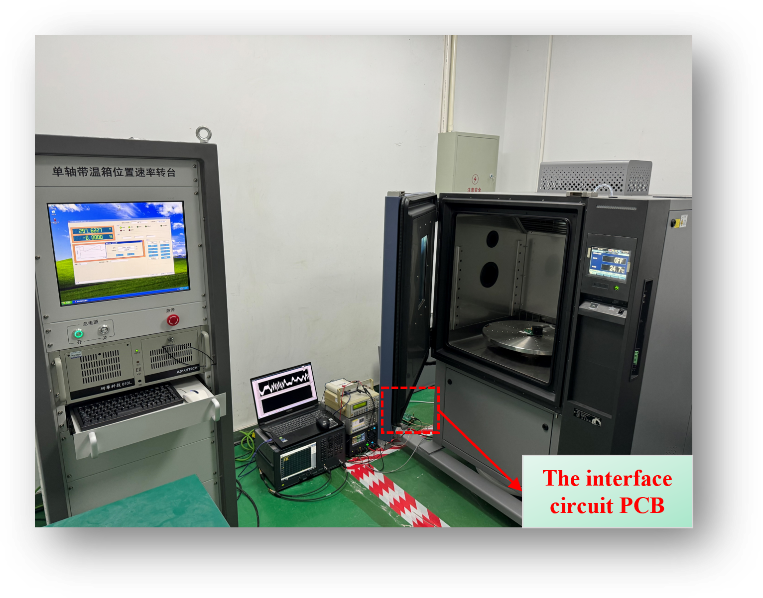


**Supplementary Fig. S9** The experimental setup for the rate sensitivity of the μHRG. The μHRG with open-loop driving and sensing circuits have been measured for the scale factor at room temperature.
